# Methane, arsenic, selenium and the origins of the DMSO reductase family

**DOI:** 10.1038/s41598-020-67892-9

**Published:** 2020-07-02

**Authors:** Michael Wells, Narthana Jeganathar Kanmanii, Al Muatasim Al Zadjali, Jan E. Janecka, Partha Basu, Ronald S. Oremland, John F. Stolz

**Affiliations:** 1 Department of Biological Sciences, Duquesne University, 600 Forbes Ave., Pittsburgh, PA 15282 USA; 2 Department of Chemistry and Chemical Biology, Indiana University Purdue University Indianapolis, Indianapolis, IN 46202 USA; 3 U.S. Geological Survey, Menlo Park, CA 94025 USA

**Keywords:** Biochemistry, Computational biology and bioinformatics, Evolution, Microbiology, Biogeochemistry

## Abstract

Mononuclear molybdoenzymes of the dimethyl sulfoxide reductase (DMSOR) family catalyze a number of reactions essential to the carbon, nitrogen, sulfur, arsenic, and selenium biogeochemical cycles. These enzymes are also ancient, with many lineages likely predating the divergence of the last universal common ancestor into the *Bacteria* and *Archaea* domains. We have constructed rooted phylogenies for over 1,550 representatives of the DMSOR family using maximum likelihood methods to investigate the evolution of the arsenic biogeochemical cycle. The phylogenetic analysis provides compelling evidence that formylmethanofuran dehydrogenase B subunits, which catalyze the reduction of CO_2_ to formate during hydrogenotrophic methanogenesis, constitutes the most ancient lineage. Our analysis also provides robust support for selenocysteine as the ancestral ligand for the Mo/W atom. Finally, we demonstrate that anaerobic arsenite oxidase and respiratory arsenate reductase catalytic subunits represent a more ancient lineage of DMSORs compared to aerobic arsenite oxidase catalytic subunits, which evolved from the assimilatory nitrate reductase lineage. This provides substantial support for an active arsenic biogeochemical cycle on the anoxic Archean Earth. Our work emphasizes that the use of chalcophilic elements as substrates as well as the Mo/W ligand in DMSORs has indelibly shaped the diversification of these enzymes through deep time.

## Introduction

Ubiquitous in *Archaea* and *Bacteria*, mononuclear molybdoenzymes of the dimethyl sulfoxide reductase (DMSOR) family are believed to have been core components of the first anaerobic respiratory chains, and thus present at life’s origins^1-4^. Reactions catalyzed by these enzymes are integral components of the carbon, nitrogen, and sulfur biogeochemical cycles, as well as the biogeochemical cycles of arsenic and selenium^5^, and likely antimony^6,7^. The family, which has been defined by the presence of a mononuclear molybdopterin or tungstopterin *bis*(pyranopterin guanine dinucleotide) (Mo/W-*bis*PGD) co-factor^5^, was named after DMSO reductases, the first members of the family to be well-characterized^8-12^.

As more representatives of the family were discovered and characterized, many were found to be heterotrimeric complexes, consisting of the catalytic subunit (the Mo/W-*bis*PGD-harboring subunit), an electron transfer subunit with as many as four [Fe–S] clusters, and a membrane anchoring subunit that tethers the complex to the membrane and transfers electrons to or from the membrane quinone pool. Thus, members of the family have also been referred to as Complex Iron-Sulfur Molybdoenzymes (CISMs)^13^. The catalytic subunit may additionally have a twin-arginine translocation motif for export to the periplasm, and a [4Fe–4S] or [3Fe–4S] iron-sulfur cluster^13,14^. It has been noted, however, that these characteristics are not uniform across the family, as there are numerous examples where one or more of the associated subunits are missing, or the catalytic subunits associate with different subunits altogether^5,13,15^. One such example is periplasmic nitrate reductase (Nap), which can be as simple as one peptide (e.g., the catalytic subunit NapA) or have additional subunits in a complex (e.g., NapABCGH)^16^.

Arsenic metabolism has previously been documented in 2.72 billion year old stromatolites, providing conclusive evidence that arsenic cycling was an active feature of Neoarchean (2.8–2.5 billion years ago (Gya)) environments^17^. This report came amidst a vigorous debate concerning the evolution of arsenic oxyanion utilization in respiration. Arsenite can serve as an electron donor to stimulate chemolithoauthotrophic growth during aerobic^18^ and anaerobic^19,20^ respiration. Arsenite can also be exploited as an electron donor to fuel photolithoautotrophic growth during anoxygenic photosynthesis^21,22^. The ability to use arsenate as a terminal electron acceptor during anaerobic respiration is a trait found among phylogenetically diverse *Bacteria* and *Archaea*^23^. Arsenate respiration is catalyzed by the respiratory arsenate reductase (Arr). An anaerobic arsenite oxidase (Arx) mediates the oxidation of arsenite during anaerobic respiration. A different enzyme, aerobic arsenite oxidase (Aio), catalyzes the oxidation of arsenite during aerobic respiration. Both Aio and Arx can function in photosynthetic organisms to exploit arsenite as an electron donor during anoxygenic photosynthesis.

The catalytic subunits of Arr and Arx, ArrA and ArxA, appear to be closely related members of the DMSOR family^20^, though both seem to be only distantly related to the catalytic subunit of Aio, AioA^18^. Phylogenies constructed using the neighbor-joining method have been used previously to support the idea that AioA was the primordial arsenite oxidase catalytic subunit and present in the last universal common ancestor (LUCA) of the two prokaryotic domains^1,24-26^, given that bacterial and archaeal homologs have formed separate monophyletic clades in these tree topologies. Presumably, chemolithoautotrophic arsenite oxidation via AioA would have been coupled to the reduction of the nitrogen oxyanions nitrate and nitrite and nitric oxide^27^, whilst the resulting arsenate would have been rapidly reduced via abiotic geochemical mechanisms. Other researchers, using geochemical and physiological arguments, have demonstrated that it is ArxA, not AioA, that couples chemolithoautotrophic arsenite oxidation to nitrogen oxyanion reduction in modern environments^21,23^, suggesting that the ArxA/ArrA lineage sustained an active arsenic biogeochemical cycle consisting of arsenite oxidation and respiratory arsenate reduction throughout the Archean Eon (~ 4.0–2.5 Gya).

This is fundamentally an evolutionary question and requires the most sophisticated tools of phylogenetic analyses in order to address it. Yet, such analyses are lacking for the DMSOR family, despite the diverse substrates utilized by DMSORs and their central role in core biogeochemical cycles. Thus, evolutionary relationships among members of this family remain poorly resolved. The only previous comprehensive phylogenetic analysis of the family employed the neighbor-joining method and lacked a root that would allow for a relative ordination of when these enzymes, and their associated biochemical functions, diversified from the broader family through geologic time^13^. Similarly, phylogenetic arguments regarding the deep antiquity of AioA employed neighbor joining phylogenies and featured unrooted topologies. More recent phylogenetic analyses of DMSOR family enzymes have exploited maximum likelihood methods, but focused on a small subset of enzymes^28,29^ and either lacked a root altogether or used enzymes within the family to root the tree.

The neighbor-joining method has long been superseded by likelihood-driven methods (e.g., Bayesian^30^ and maximum likelihood^31^ analyses) that offer more robust statistical evaluation of nodes within tree topologies as well as superior phylogenetic resolution. Moreover, recent advances in our knowledge of the probable physiology of LUCA^32^ has provided an opportunity to ordinate DMSOR phylogenies with a root that is equally as ancient as this family. To this end, the aldehyde:ferredoxin oxidoreductase family is an outstanding candidate. The aldehyde:ferredoxin family was also likely a part of LUCA’s physiology^32^ and harbors a tungstopterin co-factor^14^ similar to the DMSOR family. Finally, a wealth of genomic data has been generated since the previous comprehensive phylogenetic analysis of this family (e.g.,^33,34^), which enables much more extensive taxon sampling. The improved methodologies of likelihood-based phylogenetic methods, coupled with a wider array of genomes to mine for sequence variation, offers an ability to rigorously separate genuine vertical inheritance from LUCA from ancient lateral gene transfer events.

Taking advantage of all of these developments, we constructed maximum likelihood phylogenies consisting of 1,568 members of this family using the aldehyde:ferredoxin family as a root. We focused on a number of lineages beyond ArxA/ArrA and AioA. Phylogenetic analyses have consistently found that ArxA/ArrA is closely related to a lineage comprised of the catalytic subunits of the respiratory polysulfide reductase of *Wolinella succinogenes* (PsrA)^35^, thiosulfate reductase of *Salmonella enterica* serovar *Typhymurium* (PhsA)^36^, and selenite reductase of *Bacillus selenitireducens* (SrrA)^37^, as well as a lineage comprised of the catalytic subunits of the respiratory tetrathionate reductase of *S. eneterica* serovar *Typhymurium* (TtrA)^38^, the selenate reductase of *B. selenatarsenatis*^39^, and the arsenate reductase of the archaeon *Pyrobaculum aerophilum*^40^, whilst AioA is more closely related to formate dehydrogenase N catalytic subunits (FdhG)^1,24-26^. Rigorous phylogenetic analyses of these DMSOR members and closely related lineages has revealed several novel insights into the evolution of DMSORs. Our tree topologies robustly support a hydrogenotrophic methanogenic origin for this family, with formylmethanofuran dehydrogenase subunit B (FwdB/FmdB) constituting the most deeply branching lineage. We show that the 21st amino acid selenocysteine (Sec) is likely the ancestral ligand for the Mo/W atom of the Mo/W-*bis*PGD cofactor. Finally, our analysis marks a substantial advance in our knowledge of the evolution of arsenic respiration. We demonstrate that the ArxA/ArrA lineage is more deeply rooted in the tree topology than AioA, consistent with an Archean origin for chemo- and photolithoautotrophic arsenite oxidation in anoxic environments. Consistent with this, we show that AioA diversified from the assimilatory nitrate reductase lineage, rather than constituting a primordial enzyme present in LUCA.

## Results and discussion

### Systematics and topology of DMSOR members

**Figure 1:**
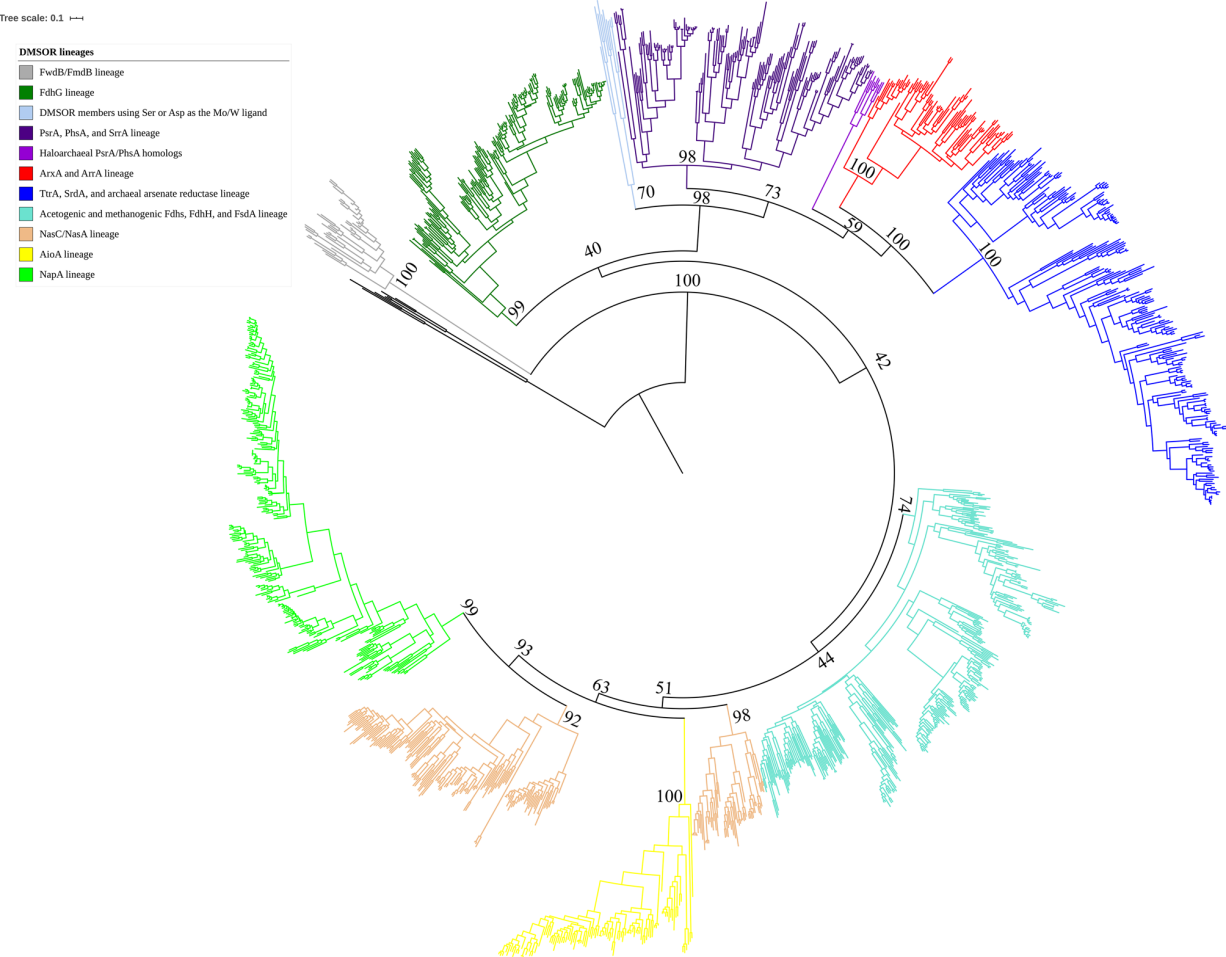
Maximum likelihood phylogeny of 1,568 DMSOR family protein sequences. All sequences came from cultured organisms with sequenced genomes. The lineage associated with each clade is indicated in the figure. The scale bar refers to the number of amino acid substitutions per site. The bootstrap support for crucial nodes in our phylogeny is provided in text.

**Figure 2:**
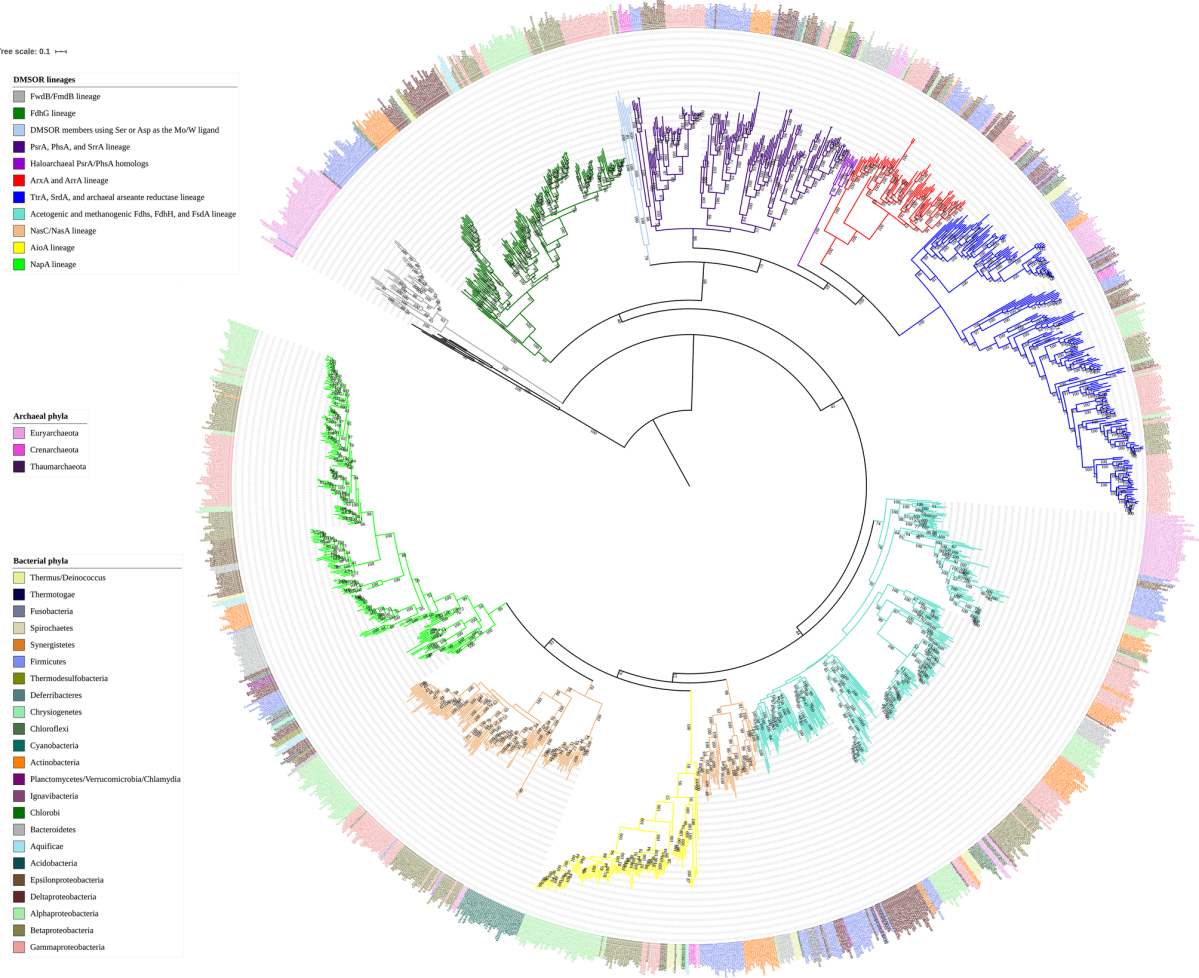
This is the same phylogeny provided in Fig. 1 with a shorthand code at each branch in the tree indicating the organism from whose genome the protein homolog was found, along with the predicted lineage of the putative DMSOR based off sequence homology to and similar operon organization with the query sequence. The key to each organism’s code is found in the Supplemental Information. The name used for each protein lineage is consistent with the rest of the text. The phylum level affiliation of each organism is indicated by the color of the text of each code name as described in the figure legend. The scale bar refers to the number of amino acid substitutions per site. Bootstrap support for all nodes is denoted in text at each respective node.

We sampled a total of fifteen physiologically distinct enzymes from the DMSOR family (Table 1) to reconstruct a maximum likelihood analysis using RAxML^41^. We produced phylogenies using untrimmed (Figs. 1, 2) and trimmed (Fig. S1) protein sequences. The phylogenies were rooted at the midpoint, which independently positioned the aldehyde:ferredoxin family as the root. Both untrimmed and trimmed phylogenies robustly supported positioning the formylmethanofuran dehydrogenase subunit B (FwdB for homologs containing W and FmdB for homologs containing Mo) within the tree topology as the most ancestral DMSOR lineage with a bootstrap support of 100. FwdB/FmdB is a member of a staggeringly complex multi-subunit formylmethanofuran dehydrogenase that was observed either as a dimer of six heterohexameric subunits or a tetramer of six heterohexameric subunits in a recently reported crystal structure^42^. While formylmethanofuran dehydrogenase catalyzes the reduction of CO_2_ and methanofuran to formylmethanofuran, the first step in hydrogenotrophic methanogenesis, structural data revealed that the FwdB/FmdB subunit specifically reduces CO_2_ to formate using ferredoxin as a physiological electron donor^42^. The crystal structures additionally confirmed that FwdB/FmdB harbors an N terminal [4Fe-4S] cluster in addition to the Mo/W-*bis*PGD cofactor, with the Mo/W atom coordinated by either a Sec or Cys residue. The subunit is composed of three domains analogous to the first three domains of formate dehydrogenases and formate dehydrogenase catalytic subunits. An additional subunit in the complex, FwdD/FmdD bears structural similarity to the fourth domain of these DMSOR members.

**Table 1:** Enzyme lineages included in the phylogenetic analysis, their function, cellular localization, and Mo/W ligand.

Mo/W-*bis*PGD catalytic subunit	DMSOR family lineage	Substrate	Function	Localization	Mo/W ligand
Polysulfide reductase (PsrA)	PsrA/PhsA/SrrA	S_n_^2−^	Reduces polysulfide in anaerobic respiration	Periplasm	Cys
Thiosulfate reductase (PhsA)	PsrA/PhsA/SrrA	S_2_O_3_^2−^	Reduces thiosulfate in anaerobic respiration	Periplasm	Cys
Selenite reductase (SrrA)	PsrA/PhsA/SrrA	SeO_3_^2−^	Reduces selenite in anaerobic respiration	Periplasm	Cys
Arsenite oxidase (ArxA)	ArxA/ArrA	AsO_3_^3−^	Oxidizes arsenite as an electron donor in anaerobic respiration or anoxygenic photosynthesis	Periplasm	Cys
Arsenate reductase (ArrA)	ArxA/ArrA	AsO_4_^3−^	Reduces arsenate in anaerobic respiration	Periplasm	Cys
Tetrathionate reductase (TtrA)	TtrA/SrdA/archaeal arsenate reductase	S_4_O_6_^2−^	Reduces tetrathionate in anaerobic respiration	Periplasm	Cys
Selenate reductase (SrdA)	TtrA/SrdA/archaeal arsenate reductase	SeO_4_^2−^	Reduces selenate in anaerobic respiration	Periplasm	Cys
Archaeal arsenate reductase	TtrA/SrdA/archaeal arsenate reductase	AsO_4_^3−^	Reduces arsenate in anaerobic respiration in some archaea	Periplasm	Cys
Formylmethano-furan dehydrogenase (FwdB/FmdB)	?	CO_2_	Reduces CO_2_ to formate in hydrogen-otrophic methano-genesis	Cytoplasm	Sec/Cys
Formate dehydrogenase N (FdhG)	FdhG	HCOO^−1^	Oxidizes formate as an electron donor in anaerobic respiration	Periplasm	Sec/Cys
NAD-dependent formate dehydrogenase	?	CO_2_	Reduces CO_2_ to formate during acetogenesis	Cytoplasm	Sec/Cys
F_420_-dependent formate dehydrogenase	?	HCOO^−1^	Oxidizes formate to CO_2_ during hydrogeno-trophic methano-genesis	Cytoplasm	Sec/Cys
Formate hydrogen lyase (FdhH)	?	HCOO^−1^	Oxidizes excess formate to carbon dioxide during fermentative growth	Cytoplasm	Sec/Cys
Arsenite oxidase (AioA)	AioA	AsO_3_^3-^	Oxidizes arsenite as an electron donor in aerobic respiration and anoxygenic photosynthesis	Periplasm	No ligand
Assimilatory nitrate reductase (NasC/NasA)	NasC/NasA	NO_3_^−^	Reduces nitrate to nitrite for assimilation into macro-molecules	Cytoplasm	Cys
Periplasmic nitrate reductase (NapA)	NapA	NO_3_^−^	Reduces nitrate to nitrite, can fulfill various physiological functions, including respiration, redox homeostasis, and assimilation	Periplasm	Cys

Question marks denote that particular DMSOR representatives have not previously been incorporated into phylogenetic analyses, thus their position within the family has not yet been resolved.

The robust placement of FwdB/FmdB as a basal lineage to formate dehydrogenases suggests that the fusion of the FwdB/FmdB and FwdD/FmdD subunits was essential for the diversification of these lineages from the DMSOR family. Formate dehydrogenases have traditionally been classified into three groups. The first group comprises a collection of periplasmic formate dehydrogenases that are canonically expressed during respiratory growth on nitrate and function to oxidize formate to CO_2_. These include the FdhG catalytic subunits of FdhN and another formate dehydrogenase (FdhO) expressed in *Escherichia coli* during respiratory growth on nitrate in low O_2_ concentrations^43^. The FdhN complex has been studied extensively (e.g.,^44,45^) but the evolutionary relationship between the FdhG catalytic subunits of FdhO and FdhN is unclear. The similar operon organization of the FdhN and FdhO complexes and high sequence identity (~ 75%) of the FdhG catalytic subunits^14^ suggest that these complexes are closely related. Consistent with this traditional taxonomy, we found that putative FdhG homologs did indeed form a coherent monophyletic clade with a robust bootstrap support of 99 in the untrimmed phylogeny and 98 in the trimmed phylogeny.

The remaining formate dehydrogenases are classified into two distinct groups, with one group comprising formate hydrogen lyases (FdhH) that functions as a member of a multi-subunit complex to reduce excess formate to CO_2_ and H_2_ during fermentative growth^43^. FdhH from *E. coli* has been extensively studied^46,47^. The other group comprises a disparate collection of NAD^+^-dependent formate dehydrogenases (FdsA) found in aerobic bacteria that function to oxidize excess formate to CO_2_, and, like FdhH, does not appear to be linked to energy conservation^48,49^. FdsA of *Cupriavidus necator* (formerly *Ralstonia eutropha*), however, can catalyze the reverse reaction (CO_2_ reduction to formate) in vitro^50^. Curiously, this taxonomy neglects the formate dehydrogenases that function in acetogenesis and hydrogenotrophic methanogenesis. The acetogenic formate dehydrogenases function, like the FwdB/FmdB subunit, to reduce CO_2_ to formate in the first step of acetogenesis, whereas the methanogenic formate dehydrogenases function to oxidize formate to CO_2_ to provide a source of CO_2_ for hydrogenotrophic methanogenesis in the presence of formate^51^_._ Our phylogeny demonstrates that classifying formate dehydrogenases by their biochemical and physiological function does not accurately reflect their evolutionary history. Acetogenic and methanogenic formate dehydrogenases, FdhH, and FdsA homologs all clustered together in a monophyletic clade with bootstrap support of 74 in the untrimmed phylogeny and 79 in the trimmed phylogeny. Thus, these various physiologically diverse formate dehydrogenases constitute members of a single evolutionary lineage.

The only substantive difference between the untrimmed and trimmed phylogenies concerns the exact positioning of the FdhG subunit within the DMSOR family. The untrimmed phylogeny positions the FdhG lineage within a clade of DMSOR members that include the respiratory dimethyl sulfoxide reductase (DmsA), respiratory nitrate reductase (NarG), PsrA/PhsA/SrrA, ArxA/ArrA, and TtrA/SrdA/archaeal arsenate reductase lineages that interact with the membrane quinone pool during anaerobic respiration using the canonical subunits identified by Rothery et al.^13^. The other formate dehydrogenase lineage clusters within a clade comprising assimilatory nitrate reductase catalytic subunits (NasA/NasC), AioA, and NapA. None of the members of this clade are associated with the canonical four [4Fe-4S] cluster electron transfer or membrane anchor subunits. While the most basal members of this clade, the acetogenic and methanogenic formate dehydrogenases, participate in energy conservation, the FdhH and FdsA homologs no longer retain this function, and the NasA/NasC subunits function exclusively in nitrogen assimilation^52^. While NapA can function as a respiratory nitrate reductase, Nap can also reduce nitrate for assimilation and redox homeostasis^16^.

This topology is consistent with the evolutionary principle of parsimony, in that it suggests that the association of DMSOR members with characteristic electron transfer and membrane anchor subunits arose once early in the evolution of DSMORs and co-evolved with these representatives through multiple diversification events. However, the trimmed phylogeny offers a topology wherein the FdhG lineage clusters with the various formate dehydrogenases, NasC/NasA, AioA, and NapA. The trimmed phylogeny also positions the FdhG lineage basal to the other formate dehydrogenases, which would suggest that components of the anaerobic electron transport chain evolved before enzyme mediated catalysis for each step of the pathways for acetogenesis and methanogenesis had fully evolved. Bootstrap support for both scenarios is weak, and thus both topologies are provided for consideration.

Both untrimmed and trimmed phylogenies position the PsrA/PhsA/SrrA, ArxA/ArrA, and TtrA/SrdA lineages as a monophyletic clade sister to DMSOR members utilizing a Ser and Asp residue to coordinate the Mo/W atom of the Mo/W-*bis*PGD cofactor. These members include NarG, DmsA, respiratory chlorate reductase catalytic subunit (ClrA), and the respiratory selenate reductase of *Thauera selenatis*^5,14^. The clade comprising the PsrA/PhsA/SrrA, ArxA/ArrA, and TtrA/SrdA/archaeal arsenate reductase lineages all utilize a Cys residue to coordinate the Mo/W atom, and also share oxidoreductase activity toward a diverse array of sulfur intermediates and arsenic and selenium oxyanions. Bootstrap support for clustering these various DMSOR members as a coherent monophyletic clade separate from the DMSOR members using Ser and Asp as Mo/W ligands is 98 in the untrimmed phylogeny and 97 in the trimmed phylogeny. The strong bootstrap support for a substantial, deeply branched clade of DMSOR members that specifically exploit chalcophiles as electron donors and terminal electron acceptors in anaerobic respiration is a powerful demonstration of the strong selective force that chalcophile utilization has exerted on the diversification of this family.

The NasC/NasA, AioA, and NapA catalytic subunits cluster with the various formate dehydrogenases, and both tree topologies suggest that this formate dehydrogenase lineage is ancestral to the NasC/NasA lineage, though bootstrap support for this is weak. However, the diversification of the various formate dehydrogenases from the family clearly predated the diversification of the archaeal and bacterial domains from LUCA, as the bacterial formate dehydrogenases form a single monophyletic clade from the substantial number of archaeal representatives (Fig. 2). This is certainly not the case for NasC/NasA. There are only two archaeal homologs present in the NasC/NasA lineage, and both were clearly inherited via lateral gene transfer, given their derived position within the lineage. The NasC/NasA and NapA catalytic subunits coordinate the Mo/W atom using a Cys residue, while the AioA subunit is the only known member of this family that doesn’t use an amino acid ligand for cofactor coordination^5,14^. The assimilatory and periplasmic nitrate reductase sequences were incorporated into our analysis when it became apparent that we could not precisely root AioA sequences within the DSMOR family without them.

**Figure 3:**
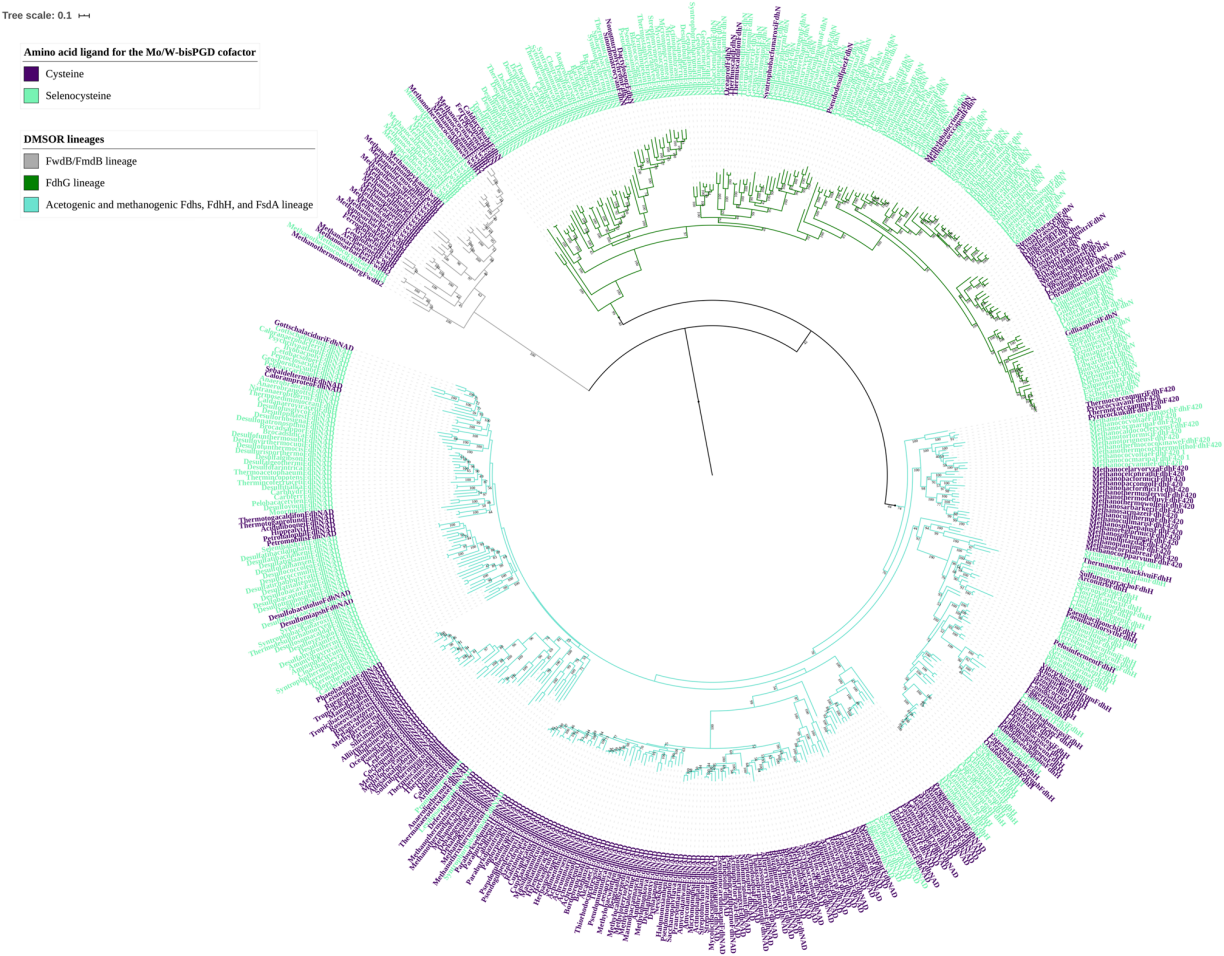
Sub-pruned portion of the maximum likelihood phylogeny showing the FwdB/FmdB, FdhG, and various formate dehydrogenase lineages. The clade for each lineage is indicated by the color of the branches. The text labels for each protein representative in the family is color coded by whether the Mo or W atom is coordinated by a Cys or a Sec residue. The scale bar refers to the number of amino acid substitutions per site. Bootstrap support for all nodes ≥ 60 is denoted in text at the respective node.

Our phylogenetic analysis revealed that these sequences were required because the AioA lineage likely diversified from NasC/NasA, as neither the untrimmed nor trimmed phylogenies could position AioA as a separate lineage from NasC/NasA with robust bootstrap support. This is in striking contrast to the NapA lineage, which clearly formed a monophyletic clade separate from NasC/NasA with a bootstrap support of 93 in the untrimmed and trimmed phylogenies. This close evolutionary relationship between NasC/NasA and AioA is quite surprising, given that such a relationship has never previously been postulated. Nevertheless, this is consistent with the physiological function of these DMSORs, which are utilized predominantly in oxic and suboxic environments. Further, this would argue for a later appearance of the NasC/NasA/AioA lineage as ammonium was the abundant form of nitrogen in the Archean^53^, obviating the need for an assimilatory nitrate reductase. A number of physiological studies have established that assimilatory nitrate reductase is expressed in both bacteria and archaea exclusively under oxic and suboxic conditions^54-58^, and in cyanobacteria assimilatory nitrate reduction occurs concomitantly with the evolution of O_2_ during oxygenic photosynthesis^59^. Likewise, AioA functions predominantly to oxidize arsenite during aerobic respiration, even in arsenite oxidizing representatives occupying the deepest branches of the lineage (Fig. 3), including representatives from the *Haloarchaea*^60^ and the *Aquificales*^61^. In fact, the only demonstration of AioA catalyzing anaerobic arsenite oxidation comes from *Chloroflexus aurantiacus*, which can exploit arsenite as a source of reducing equivalents during anoxygenic photosynthesis^22^. The potential for phototrophic arsenite oxidation exists also in certain strains of *Chlorobium limnicola* and *Chlorobium phaeobacteroides* as indicated by the presence of Aio homologs in their genomes. Many strains of *Chloroflexus* and *Chlorobium* species, however, lack Aio, indicating this ability is highly variable and a trait acquired through horizontal gene transfer^16^.

### Relative ordination of DMSOR diversifications

Our phylogenies cannot assess when, in the evolutionary record, lineages of DMSOR family enzymes diversified absent geochemical evidence for these metabolisms in dated lithologies of the fossil record. Nonetheless, the phylogeny can be used to determine whether lineages could have plausibly diverged from the DMSOR family prior to the divergence of LUCA into the *Bacteria* and *Archaea* domains. This can be assessed by determining whether homologs from the *Bacteria* form coherent monophyletic clades distinct from archaeal homologs within lineages of these enzymes. The two formate dehydrogenase lineages clearly fit this criterion (Fig. 2), particularly the lineage of physiologically diverse formate dehydrogenases which has a substantial number of representatives from the *Archaea*. It is also possible that the PsrA/PhsA/SrrA lineage diversified from the DMSOR family prior to LUCA, given that archaeal PsrA/PhsA/SrrA representatives form a coherent monophyletic clade. However, these representatives cluster with a monophyletic group of bacterial PsrA/PhsA/SrrA homologs. All of these archaeal homologs come from hyperthermophiles, and the most deeply branched bacterial homolog comes from the thermophilic bacterium *Thermosyntropha lipolytica*, which was isolated in a syntrophic coculture with a methanogenic archaeon^62^. We contend that it is likely that the PsrA/PhsA/SrrA lineage diversified from the family before the diversification of LUCA into the bacterial and archaeal domains of life. Subsequently, a lateral gene transfer event occurred between the domains, explaining the extensive vertical inheritance through many phyla observed in bacterial representatives in this clade.

The positioning of the FwdB/FmdB subunit basal to the two formate dehydrogenase lineages necessitates that this lineage diversified from the family before the diversification of the two prokaryotic domains, despite the absence of bacterial FwdB/FmdB representatives. The deep antiquity of the FwdB/FmdB and acetogenic and methanogenic formate dehydrogenase lineages is fully consistent with the hypothesis that hydrogenotrophic methanogenesis and acetogenesis represent the remnants of the earliest form of energy conservation, and that the Wood–Ljungdahl pathway represents the ancestral mechanism for carbon fixation^4,32,63^. Our phylogeny indicates that LUCA was additionally capable of energy conservation using formate as an electron donor and sulfur intermediates such as polysulfide as a terminal electron acceptor in a membrane-embedded electron transport chain.

It is curious to note that members of the three oldest DMSOR lineages have been shown to utilize Sec to coordinate the Mo/W atom of the Mo/W-*bis*PGD cofactor. This includes a number of representatives from the FwdB/FmdB lineage^64-66^, acetogenic NAD^+^-dependent formate dehydrogenases^67,68^, methanogenic F_420_-dependent formate dehydrogenases^69,70^, FdhH from *E. coli*^46^, and a number of FdhG catalytic subunits^44,45,71^. Indeed, formate dehydrogenases represent the most common selenoproteins in the genomes of Sec utilizing bacteria and archaea^72-74^. It has recently been established that the ability to synthesize and incorporate Sec into oxidoreductases was likely a feature of the metabolism of LUCA^32,75^. However, our phylogenetic analyses are the first to investigate the evolutionary history of selenoproteins (the oxidoreductases that incorporate a Sec residue) themselves. It was therefore of interest to determine if Sec use within these lineages constitutes the ancestral state. We mapped the distribution of Sec residues amongst the FwdB/FmdB, formate dehydrogenase, and FdhG lineages (Fig. 3). We found Sec utilizing homologs in each of the three lineages in deeply branched positions within the tree topology. Thus, Sec use either evolved independently in these three lineages early in their diversification or, as is more likely, Sec utilization represents the ancestral state for the earliest DMSOR representatives with the loss of Sec occurring independently at multiple points throughout all three lineages. It should be said that an ancestral sequence reconstruction analysis could statistically assess the likelihood that Sec represents the ancestral state for Mo/W coordination, but bioinformatic tools that recognize and analyze Sec residues have yet to be developed.

The ArxA/ArrA, TtrA/SrdA/archaeal arsenate reductase, NasC/NasA, AioA, and NapA lineages all unambiguously diversified from the DMSOR family after the bacterial and archaeal domains had diverged. The association of the NasC/NasA and AioA lineages with aerobic metabolism indicates that these lineages did not diversify from the family until a reservoir of O_2_ had been established on Earth surface environments. Additionally, representatives from both the AioA and the TtrA/SrdA/archaeal arsenate reductase lineages occupy some of the most recently derived branches in the tree topology. Our results starkly contradict previous findings that TtrA/SrdA/archaeal arsenate reductase^13^ and AioA^1,24-26^ represent lineages that had diversified from the family before the two prokaryotic domains had been established.

In the case of the TtrA/SrdA/archaeal arsenate reductase lineage, we demonstrate conclusively that the presence of archaeal homologs constitutes multiple lateral gene transfer events between *Bacteria* and *Archaea*. For the AioA lineage, the most ancestral homolog in the tree topology is from *B. oryziterrae*, the only member of the *Firmicutes* we found whose genome contained an AioA homolog, with a bootstrap support of 100. The second most ancestral AioA representatives comes from the haloarchaea, not the hyperthermophilic archaea, with a bootstrap support of 81. This is in contrast to the phylogenies of genuinely primordial enzymes, in which homologs from thermophilic clostridia and hyperthermophilic members of *Archaea* constitute the basal lineages of enzyme representatives for each domain, consistent with the likely hydrothermal origin of LUCA^32^. This is further buttressed by the basal position of a number of NasC/NasA representatives with respect to AioA, given that the diversification of NasC/NasA is unambiguously younger than the diversification of life into two domains.

**Figure 4:**
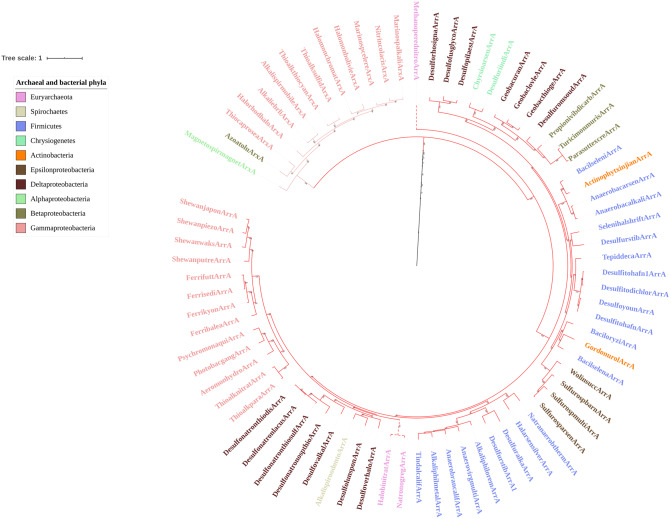
Sub-pruned portion of the maximum likelihood phylogeny showing the ArxA/ArrA lineage. The putative ArxA homologs are identified both by the text label and a lighter shade of red for the branches comprising the clade. The phylum level affiliation of each organism is indicated by the color of the text of each code name as described in the figure legend. The scale bar refers to the number of amino acid substitutions per site. Bootstrap support for all nodes ≥ 60 is denoted in text at the respective node.

The topology of the ArxA/ArrA lineage (Fig. 4) provides substantial evidence that this lineage diversified from the DMSOR family before the diversification of the AioA lineage. It has previously been proposed that ArxA homologs constitute a distinct monophyletic clade within the broader ArxA/ArrA lineage^20^. Our phylogeny decisively demonstrates that not only is this robustly supported, with a bootstrap support of 100, but that the ArxA clade is ancestral to the ArrA clade. This further undermines the hypothesis that AioA represents a primordial DMSOR member in LUCA that mediated an Archean arsenic biogeochemical cycle consisting exclusively of anaerobic arsenite oxidation. It has consistently been proposed that respiratory arsenate reduction was the initial selective pressure for the diversification of the ArxA/ArrA lineage as the Earth’s atmosphere began to accumulate O_2_ over the course of the Neoarchean and GOE^1,25,26^. Thus, ArrA should predate the diversification of the ArxA clade if anaerobic arsenite oxidation was initially mediated by AioA. The basal position of the ArxA clade instead argues that autotrophic arsenite oxidation was not a feature of LUCA, but rather evolved sometime later in the Archean. Furthermore, the greater ancestry of the ArxA/ArrA lineage with respect to AioA indicates that respiratory arsenate reduction was also widespread on the Archean Earth.

Finally, it should be noted that NasC/NasA was the only lineage in our analysis that did not form a monophyletic clade, and it could be supposed that one clade comprises NasC/NasA, whereas the other clade constitutes members misidentified as NasC/NasA. However, both clades contain biochemically and genetically characterized assimilatory nitrate reductases that we used as queries to collect sequences. This includes NasC from *B. subtilis*^76^ from the basal clade and NarB/NasA from *Synechcoccus elongatus* PCC 7942^77^ and *Azotobacter vinelandii*^78^ from the younger clade. Another potential explanation for the failure of NasC/NasA sequences to form a monophyletic clade could come from the observation that assimilatory nitrate reductases are found to have two different molecular weights, and thus some members of the lineage have sequence insertions in the primary sequence. The assimilatory nitrate reductase catalytic subunits of *B. subtilis* and *S. elongatus* PCC 7,942, for instance, are approximately 710 amino acids long and weigh approximately 70 kDa^76,79^. Proteobacterial and haloarchaeal assimilatory nitrate reductase catalytic subunits are approximately 950 amino acids long and weigh approximately 95 kDa^55,78,80,81^. A similar phenomenon has been reported for the NapA lineage, with the *Desulfovibrio desulfuricans* NapA homolog having a molecular weight of 70 kDa and the *Rhodobacter sphaeroides* NapA homolog having a molecular weight of 90 kDa^16^. These differences in the primary sequence of the assimilatory and periplasmic nitrate reductase catalytic subunits, however, are not associated with sequence insertions at specific regions of the primary sequence, but vary considerably between different taxa, and none of these sequence insertions occur at regions of the catalytic subunits essential for catalysis (e.g., the N-terminal [4Fe–4S] cluster, the Mo/W-*bis*PGD binding sites, or substate binding funnel). Thus, differences in the sequence length of NasA/NasC and NapA homologs are unlikely to explain the presence of two distinct monophyletic clades of NasA/NasC representatives. Regardless, the cyanobacterial NarB/NasA homologs cluster with the proteobacterial NasA homologs, not the *B. subtilis* NasC homologs, and the monophyly of NapA homologs was robustly supported. Finally, our phylogenetic analysis is fully consistent with a previous study of the evolution of the NapA lineage^16^, underscoring that NapA originated from NasC/NasA and that the most ancient NapA representatives come from the *Deltaproteobacteria*, *Bacteroidetes*, and *Firmicutes* phyla.

### Implications for future evolutionary study of ancient molybdo- and tungsto-enzymes

**Figure 5:**
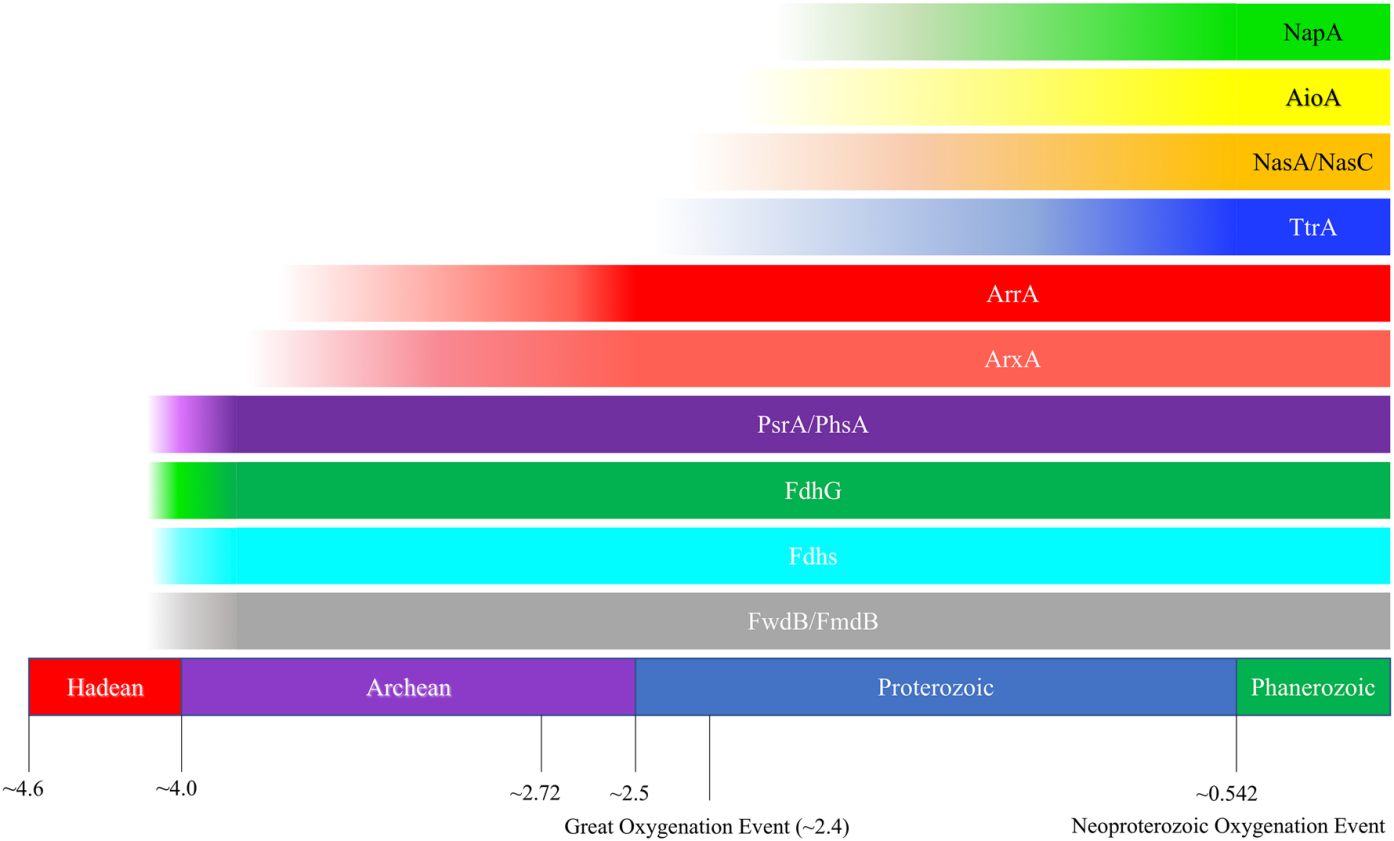
Inferred time of divergence of lineages from the DMSOR family based on our phylogenetic analysis as well as consideration of the biogeochemistry of DMSOR substrates and the physiological role (e.g., function in aerobic respiration and O_2_-dependence) of DMSOR lineages positioned over the geologic time-scale.^53^ The arsenic-containing Tumbiana stromatolites are found at ~ 2.72 Ga.^17^.

Our work has presented the first comprehensive phylogenetic analysis of members of the DMSOR family using maximum likelihood methods and appropriate outgroups to produce rooted phylogenies. This analysis has provided the first robust relative ordination of when specific lineages of DMSORs diversified from the family through geologic time (Fig. 5). We demonstrate that a sufficient phylogenetic signal exists to identify the oldest lineage of DMSOR members (FwdB/FmdB), and to provide a relative ordination for when lineages diversified from the family. Achieving this requires a substantial amount of sequence data for each lineage, as well as the most statistically rigorous phylogenetic tools available. We hope this work establishes conclusively that neighbor-joining methods are inadequate for studying this ~ 3.7 Gya assemblage of enzymes, and that appropriate roots are available for establishing a relative ordination of their diversification. Such analyses are essential for understanding the evolution of global biogeochemical cycles. We illustrate, for example, that Sec has been essential to the origin and evolution of crucial reactions in the global carbon biogeochemical cycle, including acetogenesis, methanogenesis, and carbon fixation. This underscores the deep antiquity of the global selenium biogeochemical cycle and emphasizes the importance of selenium to nascent life in catalyzing central reactions in energy conservation and autotrophic growth. We also show that while the arsenic biogeochemical cycle did not originate from LUCA, the Archean arsenic biogeochemical cycle was far more complex than originally conceived, with arsenite oxidation and arsenate reduction likely sustaining microbial communities. While our focus here was primarily on enzymes using chalcophilc elements as substrates, future analyses of other DMSOR members may further illuminate how organisms and biogeochemical cycles have co-evolved as the Earth’s oxidation state has increased over time.

## Methods

### Sequence selection

Protein representatives from DMSOR family lineages where the function of the enzyme was previously elucidated by biochemical, genetic, or structural methods were selected to be BLAST queries to construct a comprehensive library of sequences. For the families we analyzed, these included PsrA from *W. succinogenes*^35^, PhsA from *S. enterica* serovar *Typhimurium*^36^, SrrA from *B. selenitireducens*^37^, ArrA from *Chrysiogenes arsenatis*^82^ and *B. selenitireducens*^83^, ArxA from *A. ehrlichii*^19^, TtrA from *S. enterica* serovar *Typhimurium*^38^, SrdA from *B. selenatarsenatis*^39^, the arsenate reductase of *P. aerophilum*^40^, AioA from *A. faecalis*^84^ and *Rhizobium* sp. NT-26^85^, FmdB from *Methanosarcina barkeri*^86^, FmdB and FwdB from *Methanothermobacter wolfei*^87^ FdhG from *E. coli*^44^, *D. gigas*^45^, and *D. desulfuricans*^71^, FdhH from *E. coli*^46^, NAD-dependent formate dehydrogenases from *Moorella thermoacetica*^88^ and *Peptoclostridium acidaminophilum*^68^, and the F_420_-dependent formate dehydrogenases of *Methanococcus maripaludis*^70^ and *M. vannielli*^89^. These queries were blasted against the NCBI database using the DELTA-BLAST tool from the National Center for Biotechnology Information (NCBI) web server^90^.

Candidates were selected if the sequence came from an organism that had been isolated in a pure culture or defined co-culture, aligned over at least 95% of the query with an amino acid identify of at least 30%, if the sequence length was consistent with the sequence length of the query, and if the primary sequence contained motifs considered characteristic of the enzyme family (e.g., a twin-arginine translocation motif, a [4Fe–4S] or [3Fe–4S] cluster binding motif, and a Mo/W-*bis*PGD binding motif). Candidates were additionally screened using the Integrated Microbial Genomics (IMG) platform^91^ to view the genomic context of the putative homolog. Sequences were retained only if the primary sequences between the NCBI database and IMG database were conserved, and if the operon contained other subunits consistent with the operon structure described in model organisms previously (e.g., a four [4Fe–4S] cluster containing protein, a [2Fe–2S] Rieske protein, a membrane anchor).

Additional well-characterized representatives from other DMSOR family lineages were included in the analysis to provide a fuller context of where the analyzed lineages fit in the larger family. These proteins included NarG from *E. coli*^92^, *P. aerophilium*^93^, and *Thermus thermophilus*^94^, DmsA from *E. coli*^9^, chlorate reductase (ClrA) from *Ideonella dechloratans*^95^, SerA from *T. selenatis*^96^, dimethyl sulfide dehydrogenase (DdhA) from *Rhodovulum sulfidophilum*^97^, TorA from *E. coli*^98^ and *Shewanella massilia*^99^, and BisC from *E. coli*^100^. Well-characterized members of the aldehyde:ferredoxin reductase family were included as outgroups. These proteins included the aldehyde ferredoxin oxidoreductases (AORs) of *M. thermoacetica*^101^ and *Pyrococcus furiosus*^102^, the formaldehyde ferredoxin oxidoreductases (FORs) of *Pyr. furiosus*^103^ and *Thermococcus litoralis*^104^, and the glyceraldehyde-3-phosphate ferredoxin oxidoreductases (GAPORs) of *Pyr. furiosus*^105^, *M. maripaludis*^106^, and *P. aerophilum*^107^.

### Verification of selenocysteine residues in DMSOR members

Several DMSOR members, including the formyl methanofuran dehydrogenase B subunit^108^, formate dehydrogenase G subunit^44,45^, and formate dehydrogenase H^46^, have been shown to coordinate the Mo/W atom of the Mo/W-*bis*PGD co-factor with a Sec residue. This residue is located at a specific position within the primary sequence of these proteins and shares the active site of the subunits with other conserved amino acid residues (such as a neighboring His residue). These well-characterized representatives were used as query sequences in DELTA-BLAST searches for putative FmdB/FwdB, FdhG, and FdhH homologs. A putative member of these lineages was determined to be a Sec-containing homolog if the Sec residue aligned with the Sec-containing query sequence in multiple sequence alignments. Furthermore, neighboring conserved residues mentioned in X-ray crystallographic studies also had to align with query sequences before we confidently identified a putative DMSOR member as a selenoprotein. No homologs were found over the course of DELTA-BLAST searches that had more than one Sec residue in the primary sequence, or with a Sec residue that did not align with Sec residue of the active site of the well-characterized query sequences.

### Phylogenetic analysis

A total of 1,568 sequences were included for the phylogenetic analysis. The sequences were aligned using the online platform of MAFFT for large-scale sequence alignments^109^ using the G-INS-1 method. Untrimmed alignments were analyzed directly. Trimmed alignments were prepared using the trimal tool^110^. The alignments were trimmed such that all columns with gaps in more than 20% of DMSOR sequences, or with a similarity score below 0.001 were omitted, with the caveat that 60% of the columns be conserved for the analysis. The amino acid selection model that best fit our data was chosen using the ModelFinder program^111^, which compared 170 amino acid selection models. The ModelFinder program found that the LG4M amino acid substitution model^112^, which is comprised of four separate gamma distributed amino acid substitution matrices to better capture heterogeneities in mutation rates at different sites in protein alignments, was the best model for both the untrimmed and trimmed alignments. Maximum likelihood phylogenies were constructed using RAxML Version 8^41^ using the LG4M model to find the best scoring tree. The phylogenetic analysis consisted of a rapid bootstrap search followed by a search for the most likely tree topology. The autoMRE criterion was used to determine when bootstraps had converged sufficiently to use to find the most likely topology, rather than specifying a certain number of bootstraps. The model selection and maximum likelihood analyses were performed using the CIPRES gateway portal^113^. All phylogenies were visualized using Interactive Tree of Life (iTOL) platform^114^.

## Supplementary information

Supplementary file1 (DOCX 5859 kb)
