# Supplementary material for: Methane, arsenic, selenium and the origins of the DMSO reductase family
Source: Sci Rep. 2020 Jul 2;10:10946. doi: 10.1038/s41598-020-67892-9 (PMC7331816; doi:10.1038/s41598-020-67892-9)
Supplement: Supplementary file 1 — Supplementary file1 (DOCX 5859 kb) [file 41598_2020_67892_MOESM1_ESM.docx]

**Supplemental Material**

**Methane, Arsenic, Selenium and the Origins of the DMSO Reductase Family**

Michael Wells^a^, Narthana Jeganathar Kanmanii^a^, Al Muatasim Al Zadjali^a^, Jan E. Janecka^a^, Partha Basu^b^, Ronald S. Oremland^c^, and John F. Stolz^a^

^a^Department of Biological Sciences, Duquesne University, 600 Forbes Ave., Pittsburgh, Pennsylvania, 15282, USA

^b^Department of Chemistry and Chemical Biology, Indiana University Purdue University Indianapolis, Indianapolis, Indiana, 46202, USA

^c^U. S. Geological Survey, Menlo Park, California, 94025, USA

Corresponding author: John F. Stolz, 600 Forbes Ave., Pittsburgh, Pennsylvania, 15282, USA, 412 396 6333, [stolz@duq.edu](mailto:stolz@duq.edu)

**Supplementary figure legends**

**Sup. Fig. 1**

Maximum likelihood phylogeny of 1,568 DMSOR family protein sequences. All sequences came from cultured organisms with sequenced genomes. The lineage associated with each clade is indicated in the figure. The organism whose genome the protein is encoded in is provided in a shorthand code in the text at each branch, along with the lineage we predicted the protein homolog was part of based off sequence homology with the query sequence and the operon organization. The phylogeny was constructed using the RAxML program from sequences that were trimmed using the trimAL program. Bootstrap support for all nodes ≥ 40 is denoted in text at the respective node.

**Sup. Fig. 1**
